# Maternal High Estradiol Exposure is Associated with Elevated Thyroxine and Pax8 in Mouse Offspring

**DOI:** 10.1038/srep36805

**Published:** 2016-11-09

**Authors:** Ping-Ping Lv, Shen Tian, Chun Feng, Jing-Yi Li, Dan-Qin Yu, Li Jin, Yan Shen, Tian-Tian Yu, Ye Meng, Guo-Lian Ding, Min Jin, Xi-Jing Chen, Jian-Zhong Sheng, Dan Zhang, He-Feng Huang

**Affiliations:** 1 Department of Reproductive Endocrinology, Women’s Hospital, School of Medicine, Zhejiang University, Hangzhou, Zhejiang 310006, China; 2 Key Laboratory of Reproductive Genetics, Ministry of Education, Zhejiang University, Hangzhou, Zhejiang 310058, China; 3 The Center of Reproductive Medicine, the 2nd affiliated Hospital of Medical School, Zhejiang University, Hangzhou, Zhejiang 310006, China; 4 International Peace Maternity and Child Health Hospital, School of Medicine, Shanghai Jiao Tong University, Shanghai 200030, China; 5 Institute of Embryo-Fetal Original Adult Diseases and Shanghai Key laboratory of Reproductive Medicine, School of Medicine, Shanghai Jiao Tong University, Shanghai 200030, China

## Abstract

Our previous studies have shown that maternal high estradiol (E_2_) environment increased the risk of thyroid dysfunction in offspring. However, the mechanism involved remains unexplored. To evaluate the thyroid function of offspring after high E_2_ exposure and to explore the underlying mechanism, we established a high E_2_ mouse model of early pregnancy, and detected thyroid hormones of their offspring. In thyroids of offspring, the expressions of *Tg*, *Nis*, *Tpo*, *Pax8*, and *Titf1* and CpG island methylation status of *Pax8* and genes involved in methylation were analyzed. We found that thyroxine (T4) and FT4 levels of offspring were obviously increased in the high-E_2_ group, especially in females. In both 3- and 8-week-old offspring of the high-E_2_ group, *Pax8* was significantly up-regulated in thyroid glands, accompanied by the abnormal CpG island methylation status in the promoter region. Furthermore, *Dnmt3a* and *Mbd1* were obviously down-regulated in thyroids of the high E_2_ group. Besides, the disturbance of thyroid function in females was more severe than that in males, implying that the effects were related to gender. In summary, our study indicated that maternal high E_2_ exposure disturbed the thyroid function of offspring through the dysregulation and abnormal DNA methylation of *Pax8*.

The exposure to the abnormal environment in uterus may lead to chronic health problems in later life^1^,^2^,^3^, and, this impact has been observed to extend over multiple generations in both human populations and animal models^3^,^4^,^5^,^6^. High maternal estradiol (E_2_) is a significant characteristic of *in vitro* fertilization and embryo transfer (IVF-ET), and, has been suggested as an important determinative factor for the risk of small for gestational age (SGA)^7^. Accumulating evidence indicates that controlled ovarian stimulation (COH) in IVF-ET treatment might be associated with altered thyroid functions of pregnant women and rats^8^,^9^,^10^. In our previous study, we found that maternal high E_2_ environment increased the risk of thyroid dysfunction in offspring^11^. Although some studies demonstrated that increased E_2_ might alter the thyroid functions through a direct action on thyrocytes^9^,^12^,^13^, the mechanism involved in the association between the maternal high-E_2_ environment in gestational period and a high risk of thyroid dysfunction in offspring remains unclear^11^.

In mammals, epigenetic reprogramming is involved in early embryonic development^6^,^14^,^15^. Epidemiological data and animal models have demonstrated that the early life represents a window of phenotypic plasticity which is critically important for later adult metabolic health^16^. Evolutionarily and environmentally acquired genomic susceptibilities can trigger epigenomic modulations in the early life, which affect the later development of human diseases^2^,^3^,^4^. Moreover, during somatic cell development and differentiation, frequent tissue-specific and disease-specific de novo methylation events have occurred^17^. It is likely that the high E_2_ environment in early life may alter the epigenetic reprogramming and lead to the increased risks of thyroid dysfunction in offspring.

Animal models may provide the evidence of biological plausibility and potential mechanisms. To examine the effects of the high-E_2_ environment on the thyroid function of offspring, we established a mouse model with high E_2_ at the early pregnancy. We examined the expression of genes related to thyroid hormone metabolism including *Tg* (thyroglobulin), *Ni*s (Sodium-iodide symporter), *Tpo*, *Pax8* (paired box protein 8) and *Titf1* (thyroid transcription factor 1) in thyroids. Additionally, we analyzed the methylation status of CpG islands of *Pax8* in mouse thyroids to investigate whether intrauterine high E_2_ affected gene expression by regulating epigenetic modification. We further analyzed the genes involved in methylation (*Dnmt1*, *Dnmt3a*, *Tet1*, and *Mbd1*) to explore the underlying mechanisms.

## Results

### Intrauterine exposure to the high level of E_2_ altered the thyroid hormone profile in offspring

**Figure 1:**
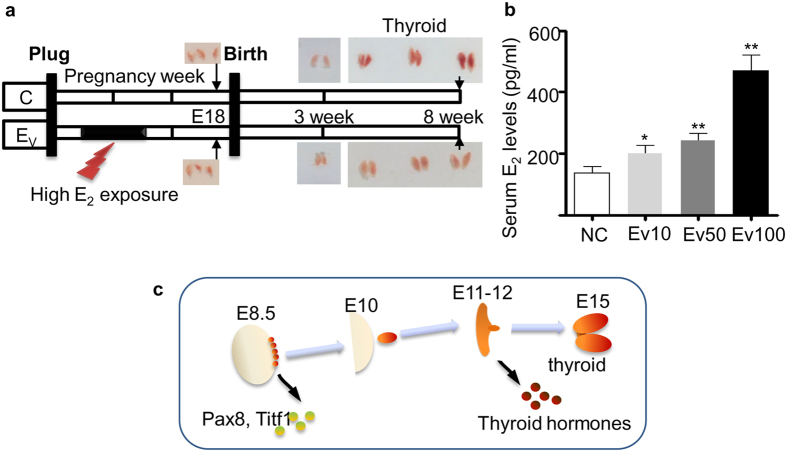
Experimental design and schematic view of thyroid morphogenesis. (**a**) Experimental design. Mice in the Ev group (Ev10/Ev100) were administrated with estradiol valerate (Ev) from day5 to day11 during pregnancy (E5-E11). (**b**) Serum E_2_ levels after Ev administration. Serum E_2_ levels were increased with dose dependence when administered with different concentrations of Ev at day11 of gestation. Results are expressed as means ± SE (n = 6). *P < 0.05 or **P < 0.01 vs. NC, student’s *t* test. (**c**) Schematic view of thyroid morphogenesis in mouse development. The thyroid gland begins to develop at day 8.5 of gestation (E8.5) as an endodermal thickening in the floor of primitive pharynx. Meanwhile, thyroid precursor cells express a combination of transcription factors. After loosing all connections with the pharynx, the thyroid bud migrates caudally, reaching its final position in front of the trachea at E10. After then, thyroid follicular cells begin their differentiative program, express thyroid-specific genes, and secrete thyroid hormones (E11-12). Finally, primitive follicles appear, and the gland displays its final morphological organization.

We established a high-E_2_ mouse model of early pregnancy and found that serum E_2_ level increased dose-dependently with the administration of estradiol valerate (Ev) (Fig. 1). We compared the serum levels of thyroid hormones among the pups conceived by NC, low-E_2_ group (Ev10) and high-E_2_ group (Ev100). The serum levels of T3, FT3, and TSH in offspring (ages: 3 and 8 weeks) from Ev10 or Ev100 group did not differ from age-matched and sex-matched offspring in the NC group (Table 1). However, the T4 levels were significantly increased in 3-week-old males and females and 8-week-old females in Ev100 group. The elevated serum FT4 levels were also observed in 3- and 8-week-old females in Ev100 group (Table 1). The abnormal thyroid hormone profile of the female offspring in high-E_2_ group seemed more obvious than that of the male offspring.

### Intrauterine exposure to the high level of E_2_ upregulated Tpo and Pax8 expression in thyroids of offspring

**Figure 2:**
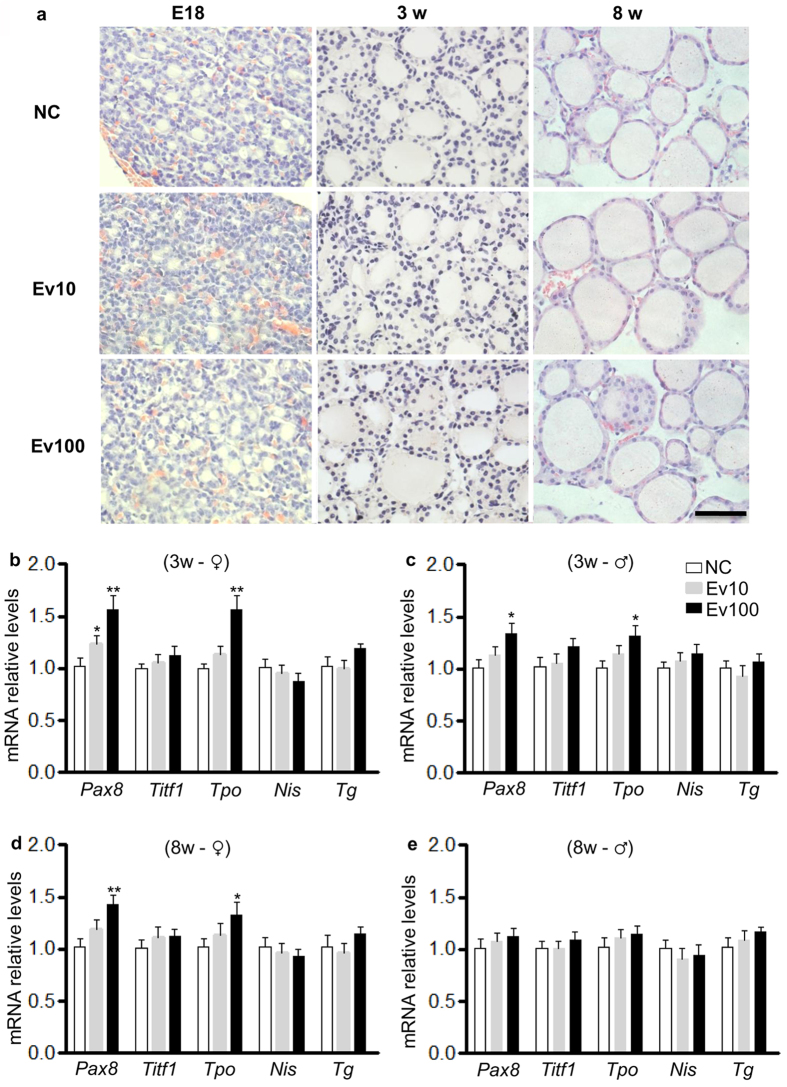
Histologic architecture and gene expression in thyroids of mice offspring. (**a**) Histologic architecture of thyroids. The histologic architecture of thyroid glands were photographed under light microscopy at E18, 3- and 8-week-old after exposure to different concentrations of E_2_ during early pregnancy. At E18, the follicular cavities were very small, and most of them had not taken shape. At 3-week-old, follicular cavities had taken shape initially and became larger; thyroid cells surrounded colloid-containing follicular cavities. At 8-week-old, follicular cavities had taken shape totally and became the largest; around the cavities, adjacent follicular cells were connected with each other by prominent terminal bars and desmosomes. No obviously difference was found among NC, Ev10 and Ev100 groups. Hematoxylin and eosin, 400×, Black scale bar, 50 μm. (**b**,**c**) Gene expression assessed by real-time quantitative PCR in thyroids of 3-week-old female (**b**) and male (**c**) pups (n = 6, respectively). (**d**,**e**) Gene expression assessed by real-time quantitative PCR in thyroids of 8-week-old female (**d**) and male (**e**) offspring (n = 6, respectively). In all panels, results are expressed as means ± SE. *P < 0.05 or **P < 0.01 vs. NC. Significance was determined by Student’s *t* test/ANOVA.

**Figure 3:**
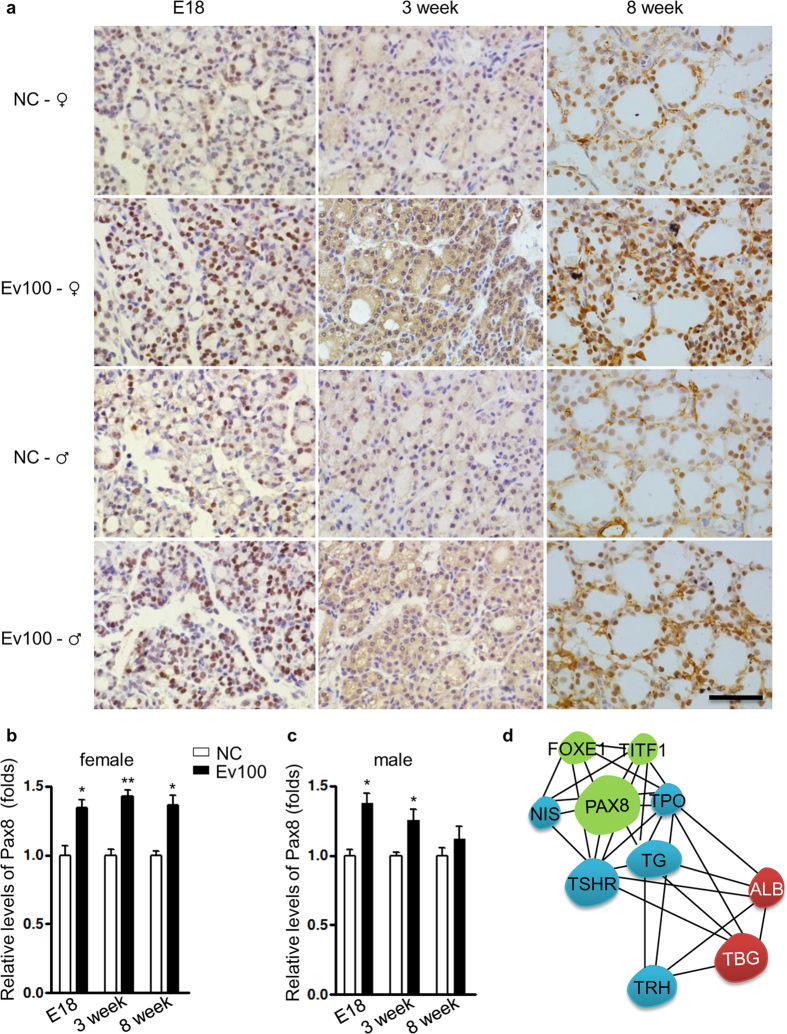
Immunohistochemical analysis of Pax8 in thyroids of mouse offspring. (**a**–**c**) Immunohistochemical analysis of Pax8 in mouse offspring. A higher immunohistochemical staining density of Pax8 was found at E18, 3- and 8-week-old of Ev100 group in female offspring (**b**); A higher immunohistochemical staining density of Pax8 was found in E18 and 3-week-old of Ev100 group in male pups, whereas no difference was found in 8-week-old male offspring (**c**). In all panels, results are expressed as means ± SE. Ev100 vs. NC, *P < 0.05 or **P < 0.01, Student’s *t* test. 400×, Black scale bar, 50 μm. (**d**) PAX8 sub-network in String database. The PAX8 association network was analyzed in String database ( http://string-db.org/), this sub-network consists of PAX8 (center) and its 9 neighbors. Green circles indicate genes of transcription factors that bind and activate the promoter of thyroid specific genes (PAX8, TITF1 and FOXE1); blue circles indicate thyroid-specific genes that directly participate in synthesis and secretion of thyroid hormones (TG, NIS, TPO, TRH); red circles indicate genes of binding and transporting thyroid hormones (TBG, ALB, TSHR); lines indicate associations between them.

**Figure 4:**
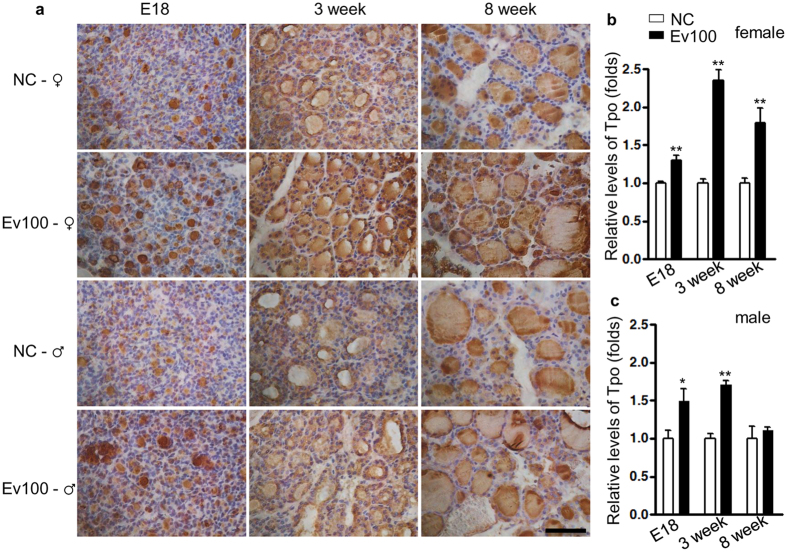
Immunohistochemical analysis of Tpo in thyroids of mouse offspring. (**a**–**c**) Immunohistochemical analysis of Tpo in mouse offspring. The expression of Tpo was significantly higher in thyroids of all E18, 3- and 8-week-old mice of Ev100 group than that of the NC group in female offspring (**b**); A higher expression of Tpo was found in E18 and 3-week-old of Ev100 group in male pups, whereas no difference was found in 8-week-old male offspring (**c**). In all panels, results are expressed as means ± SE. Ev100 vs. NC, *P < 0.05 or **P < 0.01, Student’s *t* test. 400×, Black scale bar, 50 μm.

We collected thyroids from 3- and 8-week-old mice and found no significant differences in the expression levels of *Titf1*, *Tg,* and *Nis* among groups (Fig. 2b–e). However, the mRNA levels of *Pax8* and *Tpo* in thyroids from 3-week-old males and females were both significantly higher in Ev100 group (*P* < 0.05 for males and *P* < 0.01 for females in Fig. 2b,c). In the 8th week, the expression levels of *Pax8* and *Tpo* were significantly increased in females in Ev100 group (*P* < 0.05 in Fig. 2d), whereas no significant difference was observed in males although the levels of *Pax8* and *Tpo* showed the increasing tendency in Ev100 group (*P* > 0.05 in Fig. 2e). It seems that the abnormal expression of both *Pax8* and *Tpo* of Ev100 group in female offspring is more significant than that in males. The immunohistochemical study showed that Pax8 protein was mainly located in the nucleus of follicular epithelial cells of thyroid (Fig. 3a), while Tpo protein was mainly located in the follicles and cytoplasm of follicular epithelial cells. In females, the immunohistochemical staining density of Pax8 and Tpo in E18, 3- and 8-week-old thyroids of Ev100 group was higher than that in the NC group (. In males, the higher immunohistochemical staining density of Pax8 and Tpo was found in both E18 and 3-week-old thyroids, while the difference between 8-week-old thyroids of Ev100 group and NC group was not significant (Figs 3 and 4).

### Intrauterine exposure to the high level of E_2_ induced demethylation of *Pax8* CpG island and affected Dnmt3a/Mbd1 expression in thyroids

**Figure 5:**
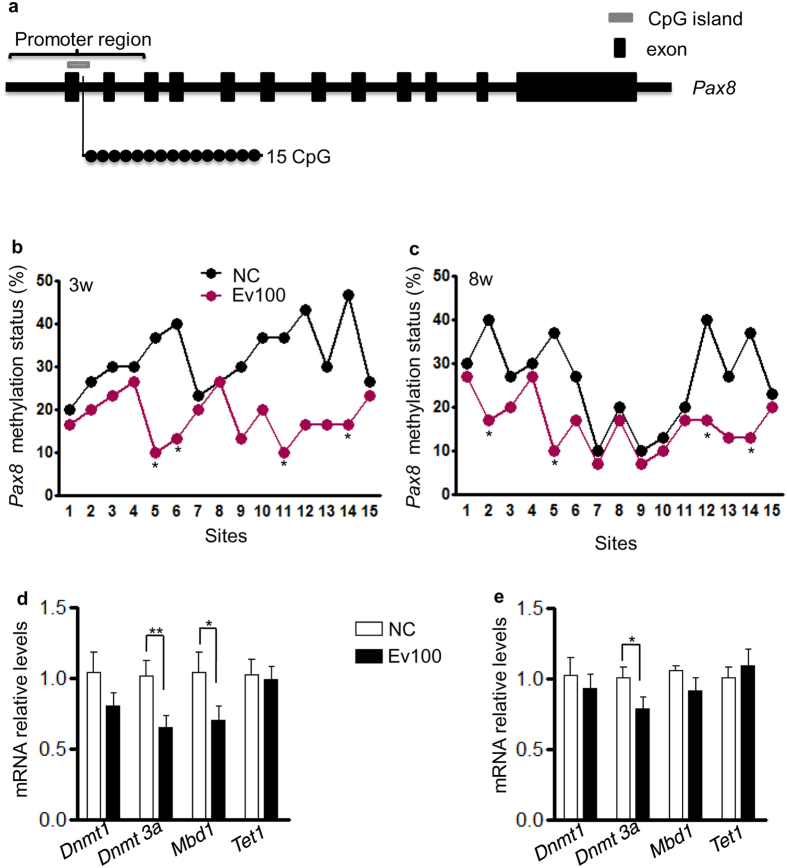
Methylation analysis of *Pax8* CpG island by bisulfite genomic sequencing PCR. (**a**) Schematic representation of mouse imprinted locus, showing the relative positions of the *Pax8* CpG island. *Pax8* CpG island containing 15 CpG sites. The positions of exons are shown as black rectangles. CpG island is indicated as grey rectangle. (**b**,**c**) Methylation status of *Pax8* CpG island and the average methylation ratio in each CpG site in thyroids of 3- (**b**) and 8-week-old (**c**). Ten clones per mouse; a total of 30 clones per group were sequenced. In the histograms, results are expressed as methylation percentage of each CpG site (n = 3 mice per group). Mean ± SE, *P < 0.05 vs. NC, x^2^ test. (**d**,**e**) *Dnmt1*, *Dnmt3a*, *Mbd1*, and *Tet1* gene expression in thyroids of 3- and 8-week-old offspring (n = 6 mice per group). Mean ± SE, *P < 0.05 or **P < 0.01 vs. NC, student’s *t* test.

**Figure 6:**
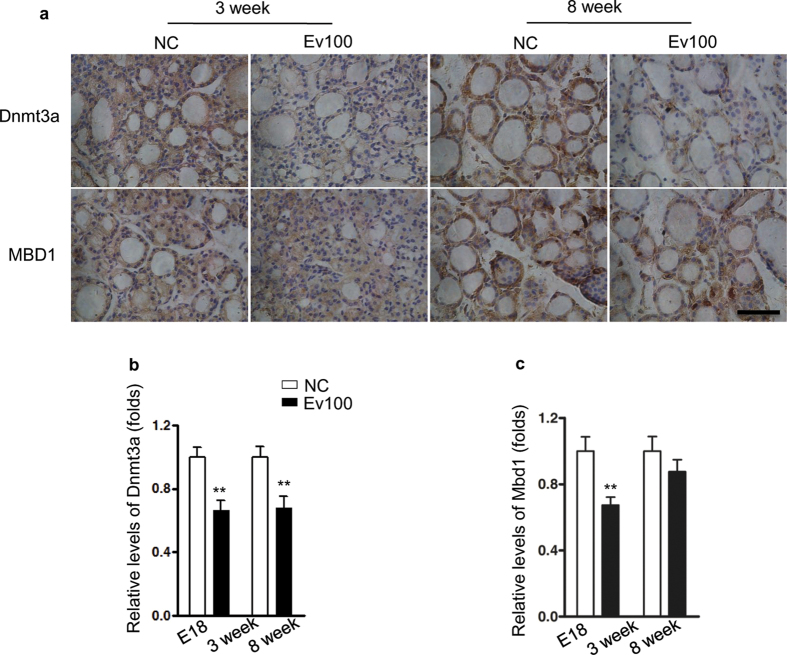
Immunohistochemical analysis of Dnmt3a and Mbd1 in thyroids of mouse offspring. (**a**) Immunohistochemical analysis of Dnmt3a in mouse offspring. The expression of Dnmt3a was significantly lower in thyroids of 3- and 8-week-old mice of Ev100 group than that of the NC group in offspring (**b**); An obviously lower expression of Mbd1 was also found in 3-week-old offspring of Ev100 group, whereas no difference was found in 8-week-old offspring (**c**). In all panels, results are expressed as means ± SE. Ev100 vs. NC, **P < 0.01, Student’s *t* test. 400×, Black scale bar, 50 μm.

We collected thyroids from 3- and 8-week-old female offspring of NC and Ev100 groups and analyzed the methylation levels of 15 cytosine phosphate guanine (CpGs) of *Pax8* CpG island in the promoter region by bisulfate sequencing PCR (Fig. 5a). We found that the Ev100 group showed significantly lower methylation levels (Fig. 5b,c). We also collected thyroids of mice offspring for quantitative real-time PCR, and found no significant difference of *Dnmt1* or *Tet1* expression between NC and Ev100 groups (Fig. 5d,e). However, *Dnmt3a* and *Mbd1* were significantly lower in Ev100 group of 3-week-old (*P* < 0.01 and *P* < 0.05, Fig. 5d). Moreover, in 8-week-old female mice, *Dnmt3a* was still significantly lower in Ev100 group (*P* < 0.05, Fig. 5e). The immunohistochemical analysis showed that the expression of Dnmt3a was significantly lower in thyroids of 3- and 8-week-old mice of Ev100 group than that of the NC group (*P* < 0.01, Fig. 6a,b). Also the lower expression of Mbd1 was found in thyroids of 3-week-old mice of Ev100 group, compared to NC group (*P* < 0.01, Fig. 6c), while there was no significant difference in 8-week-old mice between Ev100 and NC group.

### *In-vitro* exposure to the high level of E_2_ affected PAX8 and DNMT3a expression in thyroid cells

**Figure 7:**
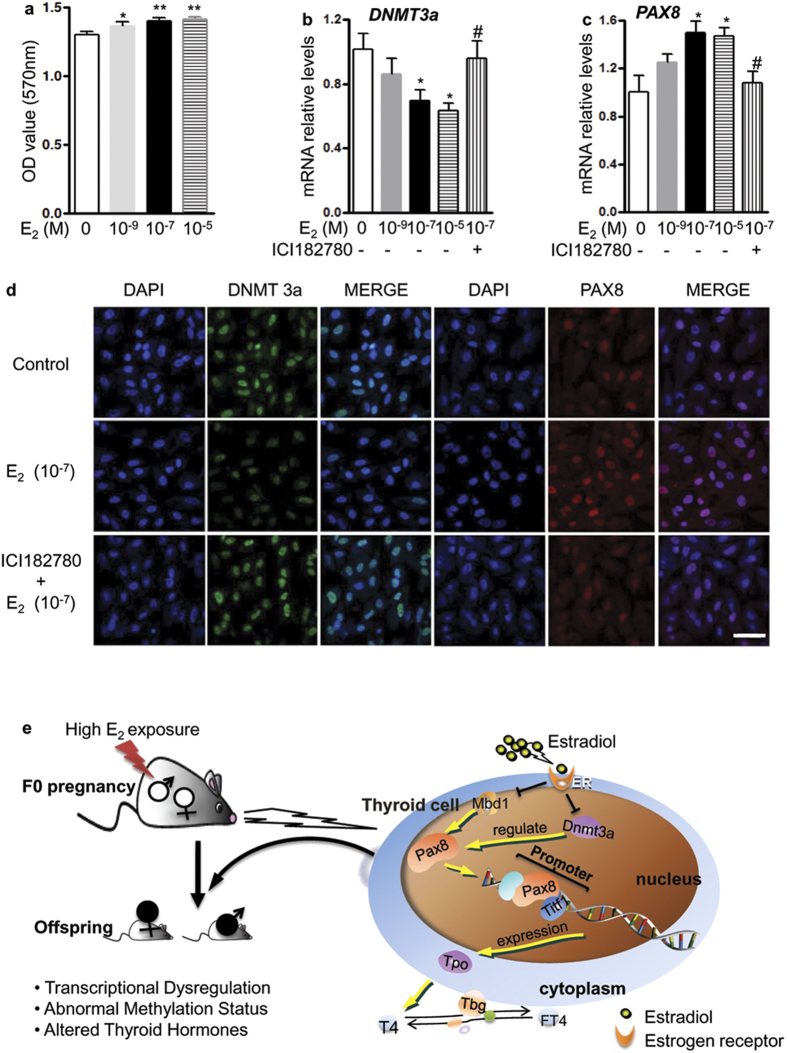
MTT assay and gene expression in nthy ori 3–1 cells. (**a**) MTT assay. Estradiol at 10^−9^ to 10^−5^ M dose-dependently increased cell proliferation (n = 6). Mean ± SE, *P < 0.05 or **P < 0.01 vs. control, student’s *t* test. (**b**,**c**) Gene expression assessed by real-time quantitative PCR in nthy ori 3–1 cells. E_2_ at 10^−7^ significantly increased *PAX8* mRNA levels (**b**) while down-regulated *DNMT3a* (**c**) in nthy ori 3–1 cells, however, its effect was blocked by pretreatment of estrogen receptor inhibitor ICI182780 (n = 4). Mean ± SE, *P < 0.05 or **P < 0.01 vs. control, ^#^*P* < 0.05 or ^##^*P* < 0.01 vs. E_2_ (10^−7^), student’s *t* test. (**d**) Immunofluorescence of DNMT3a and PAX8 in nthy ori 3–1 cells. The confocal images showed that DNMT3a and PAX8 were mainly located in the nucleus. When cells were treated with 10^−7^ M E_2_, DNMT3a was down-regulated and PAX8 was up-regulated, which were blocked by pretreatment of cells with ICI182780. White scale bar, 50 μm, mean ± SE, **P < 0.01 vs. control, ^#^*P* < 0.05 vs. E_2_ (10^−7^), student’s *t* test. (**e**) Schematic view of the possible mechanism involved in the altered thyroid hormone profile of mouse offspring after maternal exposure to high E_2_ environment. When exposed to high E_2_ levels during early pregnancy, *Pax8*/*Tpo* was up-regulated while *Dnmt3a*/*Mbd1* was down-regulated. The abnormal expression of *Dnmt3a* and *Mbd1* might alter the methylation status of *Pax8* CpG islands and increase the expression of *Pax8,* a transcription factor that binds and activates the promoter of thyroid specific genes such as *Tpo,* in offspring, so the synthesis/secretion of thyroid hormones increase. Finally, pups displayed altered thyroid hormones companied with abnormal gene expression and changed methylation status.

In order to verify the direct effect of high E_2_ on thyroid development, nthy-ori3–1 cells were cultured for 24 h in the medium containing different concentrations of E_2_. We found that E_2_ in the concentration range of 10^−9^ to 10^−5^ M increased cell proliferation (Fig. 7a). Meanwhile, E_2_ at 10^−7^ and 10^−5 ^M significantly reduced *DNMT3a* and increased *PAX8* expression compared with the control group (*P* < 0.05, Fig. 7b,c). The down-regulated *DNMT3a* or up-regulated *PAX8* was blocked by pretreating cells with estrogen receptor inhibitor ICI182780 (10^−5^ M), indicating that the estrogen receptor was involved. The confocal images showed that DNMT3a and PAX8 were mainly located in the nucleus (Fig. 7d). When cells were treated with 10^−7^ M E_2_, DNMT3a was obviously down-regulated, while PAX8 was significantly up-regulated (Fig. 7d). The above two regulation effects were blocked by pretreating cells with ICI182780.

## Discussion

Suboptimal maternal environment during pregnancy predisposes offspring to chronic diseases in later life^18^, and the early implantation period is also a critical stage for the development and later adult health^2^,^19^. Human epidemiological investigation indicated a link among mothers exposed to famine in utero with LBW in their offspring^20^. Animal models showed that maternal undernutrition during pregnancy could induce IGT and obesity in offspring^5^. Children conceived by IVF might have the increased risk of developing metabolic syndrome^21^,^22^. In previous studies, we found that the environment of high E_2_ level was correlated with the increased risks of LBW and SGA, which might lead to chronic diseases in later life^7^.

In our study, after exposure to the high E_2_ level in early pregnancy, abnormal secretion of thyroid hormones was found in their offspring. In the 3rd week, in both males and females, the levels of T4 were significantly increased in Ev100 group; meanwhile FT4 levels were significantly increased in female pups. Importantly, T4 and FT4 levels were still significantly increased in 8-week-old female offspring of Ev100 group, but there was no difference in male offspring. Actually, the expression of Pax8 and Tpo in thyroids of Ev100 offspring was up-regulated, suggesting that the environment of the high E_2_ level during early pregnancy might lead to the dysfunction of thyroid cells in offspring. *In-vitro* experiments also verified the increased *PAX8* expression after treating thyroid cells with the high E_2_ dose. Abnormal expression of the genes related to the thyroid function induced by the high E_2_ dose might affect the secretion of thyroid hormones. Moreover, our results proved that E_2_ could dose-dependently increase proliferation of Nthy-ori 3–1 cells, indicating a direct effect of this hormone on thyroid cells.

Many factors are related to the dysfunction of thyroid cells. In this study, we focused on some genes involved in thyroid development and functions including *Tpo*, *Tg*, *Nis*, *Pax8*, and *Titf1*. TPO is involved in iodinating and coupling of the hormonogenic tyrosines in TG to yield the thyroid hormones T3 and T4^23^. PAX8 and TITF1, known as the transcription factors that bind and activate the promoter of thyroid specific genes, are involved in maintaining the functional differentiation of thyroid cells^24^. Bioinformatics analysis shows that PAX8 can affect other transcription factors that bind and activate the promoter of thyroid specific genes (TITF1 and FOXE1). Moreover, PAX8 is related to thyroid-specific genes that directly participate in synthesis and secretion of thyroid hormones (TG, NIS, TPO, and TRH) and influence the genes of binding and transporting thyroid hormones (TBG, ALB, and TSHR) (Fig. 3d). Finally, the up-regulated expressions of *Pax8* and *Tpo* were exhibited in offspring of high E_2_ dose group, confirming that the dysregulation of *Pax8* and *Tpo* led to inappropriate production and secretion of thyroid hormone. Similar to the changing tendency of thyroid hormone, the change of gene expression was also more obvious in female offspring than in males. The results indicated that the intrauterine exposure to high E_2_ dose could change gene expression of thyroid cells and alter the thyroid hormone profile. DNA methylation could control gene expression by modulating enhancer access to the gene promoter through the regulation of an enhancer boundary. We further analyzed the promoter region of *Pax8* and *Tpo* with UROGENE and found a CpG island in *Pax8*, but no CpG island was found in *Tpo*. Because thyroid hormone profile and gene alteration were both more obvious in female offspring, we examined the *Pax8* CpG islands in thyroid glands of female pups. We found the methylation levels in *pax8* CpG islands were significantly lower in thyroids of 3- and 8-week-old Ev100 groups. These results indicated that intrauterine high E_2_ could alter the methylation status of *Pax8* promoter in offspring and that the altered expression of *Pax8* in thyroids was associated with the altered modification of *pax8* CpG islands. Because Pax8 is a transcription factor that binds and activates the promoter of thyroid specific gene *Tpo*, and the upregulation of Pax8 may be fully or partly responsible for the abnormal Tpo expression. Further studies are also required in order to better understand the mechanisms of the higher Tpo levels found in Ev100 pups.

Additionally, we focused on the genes involved in DNA methylation: *Dnmt1*, *Dnmt3a*, *Mbd1*, and *Tet1*. Dnmt1 predominantly catalyzes maintenance methylation^25^. Dnmt3a are mainly responsible for de novo methylation that establishes a new DNA methylation state at repeated sequences, developmental genes, and imprinted genes^26^. Tet1 are dioxygenases and plays a key role in active DNA demethylation^27^. Mbd1 protein is one of the components of MeCP1, and plays a role in methylation-mediated transcriptional silencing in euchromatin^28^. Although the difference in *Dnmt1* or *Tet1* expression was not significant, down-regulated expression of *dnmt3a/Mbd1* was observed in 3-or 8-week-old pups of Ev100 group, suggesting that the dysregulation of *dnmt3a* and *Mbd1* might be involved in hypomethylation of *pax8* CpG island. *In vitro* cultivation confirmed the effect of high E_2_ dose on *Dnmt3a* expression in nthy-ori 3–1 cells, providing an explanation for the direct influence of intrauterine high E_2_ dose on *Dnmt3a* gene expression and on altering the methylation status in thyroids of fetus. The possible mechanism of the altered thyroid hormones after exposure to the high E_2_ environment is summarized in Fig. 7e.

Surprisingly, the alteration of thyroid hormones in Ev100 female offspring was more significant than that of male offspring, suggesting that the thyroid function of females might be more sensitive to the intrauterine environment of the high level of E_2_ than males. A number of thyroid disease-related animal models showed gender differences, with females having a more profound phenotype^29^, but the molecular basis for this gender-based difference is still poorly understood. Many previous studies have linked E_2_ action to the increased prevalence of the thyroid disease in females^30^,^31^,^32^,^33^. In the Pten knockout mice, a significant gender difference was obtained: almost 52% of Pten^−/−^ females showed the developed follicular adenomas while only 12% of Pten^−/−^ males were affected^31^. In our study, the intrauterine exposure to high E_2_ dose not only reveals its role in thyroid disorders, but also sheds light on the molecular basis of this gender-based difference. Even conceived by natural conception, compared with the non-pregnant status, the serum E_2_ levels during gestation were extremely higher. Perhaps, imprinting of some gene occurred and epigenetic status altered after exposure to the environment of the high E_2_ when thyroid was differentiating and developing. Moreover, the sensitivity to the environment of the high E_2_ dose might be varied among individuals. Because female pups have more estrogen receptors in fetus and more estrogen secretion after birth than males even in the childhood^34^, the difference between them becomes more apparent when pups grow up. Further studies are required in order to better understand the mechanisms involved.

In conclusion, with an intrauterine high-E_2_ mouse model, we found that high serum E_2_ level during early pregnancy induced the abnormal thyroid hormone levels in offspring, especially in females, which were partly caused by the abnormal expression of the genes involved in thyroid development and function. The expression abnormality could be attributed to the dysregulation of methylation status caused by the altered expression *of dnmt3a* and *Mbd1* genes. Thus, we demonstrated, for the first time, that thyroid hormones, gene expression and methylation status in thyroids were altered by E_2_ administration during early pregnancy. Additionally, this impact was more obvious in female offspring, indicating that the higher incidence of the thyroid disease in females might be partly ascribed to the estrogen exposure in uterine. As controlled ovarian hyperstimulation (COH) of ART situates the gamete/fetus in a supra physiological E_2_ environment, our findings provide new insights into the security of IVF children and underline the importance of continuous monitoring of endocrine axes of those children who are exposed to the environment of high E_2_ dose during early pregnancy. Further studies are required in order to confirm these findings and better determine their etiopathogenesis and clinical significances.

## Methods

All animal protocols were reviewed and approved by the Zhejiang University Animal Care and Use Committee. The methods were carried out in accordance with the relevant guidelines, including any relevant details. The estrous cycle was evaluated by daily vaginal cytology, and all the adult female mice included in the study had regular cycle for at least 2 weeks before the experiments. At the age of 8 weeks, virgin female ICR mice (n = 60) were mated with normal males (n = 30). Onset of pregnancy was determined by the presence of a copulation plug after overnight mating (designated as day 0 [E0] of pregnancy). Afterwards, the females were randomly divided into the control group, low E_2_ group (Ev10) and high E_2_ group (Ev100). Mice in the Ev10 group were oral administrated with estradiol valerate (Ev, Sigma, St. Louis, MO) which was dissolved in cornoil at a dose of 10 μg/kg/day while Ev100 group at a dose of 100 μg/kg/day from day5-11 (E5-E11). Control pregnant females received an equal volume of cornoil. In order to verify the serum E_2_ level after Ev administration, other 24 females, who were divided into 4 groups and administrated with E_2_ at 0, 10, 50, and 100 μg/kg per day, respectively, were sacrificed on day11 of pregnancy, and, their blood was collected for measurement of the serum E_2_ concentrations. The pregnant mice were allowed to deliver spontaneously. The litter size was randomly reduced to 8 at birth to assure uniformity.

### Thyroid collection

Thyroid glands were segregated from the thyroidcartilage of fetal day 18 (E18), 3-week-old and 8-week-old adult mice, respectively. The thyroids were washed in phosphate-buffered saline (PBS) and stored in liquidnitrogen (−170 °C).

### *In vivo* serum levels of E_2_ and thyroid hormones

Control and Ev administrated female mice were sacrificed at day11 of pregnancy to detect the serum E_2_, and, blood specimens of 3- and 8-week-old mouse offspring were collected to detect thyroid hormone levels. Serum E_2_ levels were analyzed by an EIA method (estradiol EIA kit, Cayman, MI, catalog # 582251). Serum T3, FT3, T4, FT4 and TSH levels were assayed with the RIA counter (XH6080, Xi’an Nuclear Instrument Factory) by Beijing North Institute of Biological Technology (Beijing, China). The intra- and inter-assay coefficients of variation for the determination of all biochemical variables were less than 10%.

### Gene expression (Quantitative PCR)

Total RNA was extracted from cells or tissues using the Trizol reagents and reverse-transcribed method according to manufacturer’s instructions. Quantitative real-time PCR analysis was carried out in an Applied Biosystems 7900 Fast (Applied Biosystems, Foster City, CA). *TBP* or *β-2mg* served as internal control. The primer sequences used to amplify *Pax8, Titf1, Tpo, Nis, Tg, Dnmt1, Dnmt3a, Mbd1* and *Tet1* are listed in Supplemental Table S1.

### DNA methylation (bisulfite genomic sequencing PCR)

Genomic DNA was extracted from thyroids of 3 and 8-week-old mice in the control and Ev100 groups. EpiTect bisulfite kit (Qiagen, Valencia, CA) was used to deaminate cytosine to uracil according to the manufacturer’s instructions, while 5-methyl-cytosine was protected from deamination. Nested PCR was used to increase the specificity of DNA amplication (Primers are listed in Supplemental Table 1). Methylation status of the *Pax8* CpG island was determined by cloning and sequencing of bisulfite-treated DNA. The purified PCR products were cloned using the pMD19-T vector system (TaKaRa, Dalian, China). The sequence obtained by cloning was analyzed with 3730 DNA Analyzer polymers (Applied Biosystems, Carlsbad, CA).

### Hematoxylin-eosin (HE) assay and immunohistochemical analysis

The endometrial samples embedded in paraffin were sectioned at 4 μm. For HE assay, slides were prepared and stained with hematoxylin and eosin. The analysis was performed with an optical microscope with magnification of 400 times. For immunohistochemical analysis, slides were blocked in 5% BSA, and incubated with a 1:100 dilution of primary antibody against Pax8 (Abcam, San Francisco, CA, ab53490), Tpo (Santa Cruz, CA, sc-376876), Dnmt3a (Santa Cruz, CA, sc-20703) or Mbd1(Santa Cruz, CA, sc-10751). After incubation with secondary antibody, the sections were reacted with diaminobenzidine (Dako Cytomation, Carpinteria, CA), counterstained with hematoxylin, dehydrated, and mounted in distrene dibutylphthalate xylene.

Immunostained sections were characterized quantitatively by digital image analysis with Image Pro-Plus 6.0 according to the method introduced by Wang-Tilz *et al.*^35^. Images were obtained with a Nikon ECLIPSE Ti-S microscope fitted with a microimage video camera. A series of 10 random images on several sections were taken for each immunostained parameter to obtain a mean value for statistical comparison. Staining was defined by color hue and intensity, and the area with color hue and intensity in a certain range was chosen as the positive area. The selected positive area regimen was then applied equally to all images to create preview images and measurements were obtained. The intensity of the labeling was determined by the computer program and gave a gray value ranging from zero (black) to 256 (white). Immunohistochemical parameters assessed in the area include the mean intensity. All sections were inspected independently by three observers. All the data were presented as the fold change in protein expression normalized to the control.

### Cell culture

Nthy-ori 3–1 cells (HPACC, Salisbury, UK), originated from normal human thyroid cells which had been immortalized by the expression of SV40 large T antigen, were cultured in phenol red DMEM medium supplemented with 10% fetal bovine serum (FBS; vol/vol) at 37 °C plus 5% CO_2_.

### Cell proliferation assay

The MTT assay was applied to quantify the cell proliferation. The cells were incubated with 0.5 mg/ml methyl thiazolyl tetrazolium (MTT) for 4 h. The formazan crystals produced by the living cells in the culture were dissolved with 100 ml dimethylsulfoxide, and the absorbance (OD value) was measured at 570 nm using a 96-well plate reader at 24 h.

### Immunofluorescence analysis

Approximately 10^4^ nthy-ori 3–1 cells were grown on coverslips and exposed to treatments. The fixed and permeabilized cells were blocked in 5% BSA and then incubated with a 1:100 dilution of primary Pax8 antibody (Abcam, San Francisco, CA, ab53490) and Dnmt3a antibody (Santa Cruz, CA, sc-20703) at 4 °C overnight. They were incubated with a 1:200 dilution of Alexa Fluor594 goat anti-mouse IgG and Alexa Fluor488 goat anti-rabbit (Invitrogen, Carlsbad, CA) for 2 h. The slides were analyzed with an Olympus BX51TF fluorescence microscope (Olympus Corporation, Tokyo, Japan).

### Bioinformatics analysis

The Pax8 association network was analyzed in String database ( http://string-db.org/).

### Statistical analyses

Data are presented as means ± SE. Statistical analyses were performed by two-tailed Student *t* test, one-way ANOVA, or *x*^*2*^ test (version 16.0, SPSS) as described in the table and figure legends. *P* < 0.05 was considered statistically significant.

## Additional Information

**How to cite this article**: Lv, P.-P. *et al.* Maternal High Estradiol Exposure is Associated with Elevated Thyroxine and Pax8 in Mouse Offspring. *Sci. Rep.* **6**, 36805; doi: 10.1038/srep36805 (2016).

**Publisher’s note:** Springer Nature remains neutral with regard to jurisdictional claims in published maps and institutional affiliations.

## Supplementary Material

Supplementary Information

## Figures and Tables

**Table 1:** The profile of thyroid hormones in mouse offspring born after NC and high E_2_ exposure during early pregnancy.

	3 weeks			8 weeks		
	NC (n = 7)	Ev10 (n = 6)	Ev100 (n = 6)	NC (n = 7)	Ev10 (n = 6)	Ev100 (n = 6)
*female*						
T3 (ng/ml)	0.77 ± 0.08	0.78 ± 0.12	0.78 ± 0.13	0.87 ± 0.03	0.86 ± 0.06	0.87 ± 0.04
FT3 (fmol/ml)	2.77 ± 0.33	2.71 ± 0.29	2.69 ± 0.43	2.76 ± 0.18	2.72 ± 0.25	2.99 ± 0.22
T4 (ng/ml)	45.68 ± 4.08	49.36 ± 3.12	63.74 ± 5.41**	49.92 ± 2.93	53.38 ± 2.52	61.68 ± 2.55*
FT4 (fmol/ml)	11.43 ± 1.11	13.20 ± 1.16	15.93 ± 1.28*	11.63 ± 0.70	12.58 ± 0.63	14.37 ± 0.52*
TSH (mIU/L)	1.81 ± 0.11	1.71 ± 0.13	1.68 ± 0.11	1.84 ± 0.15	1.76 ± 0.16	1.69 ± 0.13
*male*						
T3 (ng/ml)	0.61 ± 0.06	0.63 ± 0.09	0.61 ± 0.09	0.92 ± 0.04	0.88 ± 0.05	0.82 ± 0.03
FT3 (fmol/ml)	3.47 ± 0.34	3.49 ± 0.24	3.52 ± 0.26	2.69 ± 0.31	2.73 ± 0.29	2.55 ± 0.13
T4 (ng/ml)	50.32 ± 2.46	52.67 ± 2.75	57.98 ± 3.97*	49.91 ± 2.27	51.22 ± 2.06	52.34 ± 3.89
FT4 (fmol/ml)	12.03 ± 1.16	12.65 ± 1.08	13.62 ± 0.71	12.45 ± 0.67	13.12 ± 0.69	12.73 ± 1.23
TSH (mIU/L)	1.89 ± 0.13	1.81 ± 0.09	1.78 ± 0.15	1.91 ± 0.11	1.83 ± 0.15	1.89 ± 0.12

Data are shown as mean ± SE, ^*^P < 0.05 or ^**^P < 0.01 vs. NC.
