# Supplementary material for: Maternal High Estradiol Exposure is Associated with Elevated Thyroxine and Pax8 in Mouse Offspring
Source: Sci Rep. 2016 Nov 9;6:36805. doi: 10.1038/srep36805 (PMC5101534; doi:10.1038/srep36805)
Supplement: Supplementary Information [file srep36805-s1.doc]

**Maternal High Estradiol Exposure is Associated with Elevated Thyroxine and Pax8 in Mouse Offspring**

Ping-Ping Lv, Shen Tian, Chun Feng, Jing-Yi Li, Dan-Qin Yu, Li Jin, Yan Shen, Tian-Tian Yu, Ye Meng, Guo-Lian Ding, Min Jin, Xi-Jing Chen, Jian-Zhong Sheng, Dan Zhang and He-Feng Huang

Table S1. Primers for Nested-PCR and qPCR (SYBR Green).

| cDNA | Primer set | Sense primer(5’-3’) | Antisense primer(3’ -5’) | ACCESSION |
| --- | --- | --- | --- | --- |
| h*PAX8* |  | GGTGGTGGAGAAGATTGGG | GGGACTCAGGGACTTGGTG | NM_003466 |
| h*DNMT3a* |  | TATTGATGAGCGCACAAGAGAGC | GGGTGTTCCAGGGTAACATTGAG | NM_175629 |
| h*TBP* |  | TGCACAGGAGCCAAGAGTGAA | CACATCACAGCTCCCCACCA | NM_003194 |
| m*Pax8* |  | CACAAAGGCCCCTCCTAGTT | GCGAGTGTCCCTCAGTCTGT | NM_011040 |
| m*Titf1* |  | AGCGGCATGAATATGAGTGGC | TGCGTGGGTGTCAGGTGAA | NM_009385 |
| m*Tpo* |  | TGTCCAGTGTCTTGAGCTGC | TGCCGATCACAGAGAGAGTG | NM_009417 |
| m*Nis* |  | CCCAGAGCCCCGTAGTAGAG | AGCTGCCAACACTTCCAGAG | NM_053248 |
| m*Tg* |  | GGCCCTCTCTGGGCTGATA | GTGGCCAGCATACACCTTCT | NM_009375 |
| m*β-2mg* |  | TTCAGTATGTTCGGCTTCCC | TGGTGCTTGTCTCACTGACC | NM_009735 |
| m*Dnmt1* |  | GCCATCTCTTTCCAAGTCTTT | TGTTCTGTCGTCTGCAACCT | NM_001199431 |
| m*Dnmt3a* |  | ACTCTCCAGAGGCCTGGTTC | TACATCAGCAAACGGAAACG | NM_007872 |
| m*Mbd1* |  | TATGTAGCCTTGGCAACCAG | GAGGAGATAGGAGGGGTTGG | NM_013594 |
| m*Tet1* |  | CTAGGTTTGGCCAGAAGGGG | ACAGGTGCAGGTACGCTTTT | NM_001253857 |
| *m*Pax8* | Outer set | GAGGGGATGTGGGTTTGA | CCCCACAAATAATCCCTCACTA | 18510 (Gene ID) |
|  | Inner set | TGGGTTTATATGTAGGGTAGT | TCCTACCAACTTAAAATTTTCTCAC |  |

*Nested PCR.
